# Measurement of Nontechnical Skills During Robotic-Assisted Surgery Using Sensor-Based Communication and Proximity Metrics

**DOI:** 10.1001/jamanetworkopen.2021.32209

**Published:** 2021-11-02

**Authors:** Jackie S. Cha, Dimitrios Athanasiadis, Nicholas E. Anton, Dimitrios Stefanidis, Denny Yu

**Affiliations:** 1 Purdue University School of Industrial Engineering, West Lafayette, Indiana; 2 Department of Industrial Engineering, Clemson University, Clemson, South Carolina; 3 Department of Surgery, Indiana University School of Medicine, Indianapolis

## Abstract

This cohort study uses sensor-based communication and proximity metrics to assess surgeon nontechnical skills during robotic-assisted surgery.

## Introduction

Nontechnical skills (NTS) include cognitive and interpersonal (eg, teamwork) skills^1^; they are linked to patient outcomes in surgery.^2,3^ Currently, NTS assessment in surgery relies on behavior ratings that are subjective, potentially biasing assessments, and requires trained raters, a limited resource. This study demonstrates a sensing-based system for objectively measuring intraoperative NTS. We hypothesized that objective, sensor-derived communication and proximity metrics are associated with NTS scores assessed with existing tools and can accurately predict exemplar NTS scores that promote patient safety and teamwork.

## Methods

This cohort study, conducted between January 1 and December 31, 2019, was approved by the Indiana University institutional review board and followed the Strengthening the Reporting of Observational Studies in Epidemiology (STROBE) reporting guideline for observational studies. No race or ethnicity data were collected because those details might identify participants. The Non-Technical Skills for Surgeons (NOTSS) tool with reported validity evidence was used as the gold standard NTS assessment. Two raters assessed surgeon NTS, and interrater reliability was determined.^4^ Three categories of objective metrics were captured with wearable sensors and video (Table): communication, speech (Praat version 6.1.01), and proximity of surgical team member location (Figure).

**Table.** Objective Metrics From Communication, Speech, and Proximity Sensors

Variable	Description or definition		Example
**Communication metrics (captured with lapel microphones and video)**			
Type^a^			
Request	Requesting, directing, or instructing an individual to complete an action		“Pass me the suture.”
Confirmation	Verifying or confirming a statement or that an action was acted on		“Yes, that is correct.”
Question	Asking an individual about a value, state, or action		“Do you see any bleeding?”
	If a statement was asking for a confirmation (eg, “Is this the correct patient?”), it was classified as a question		
Goal sharing/status	Sharing information to create understanding of current state or expectation of future state		“We’re ready to dock the robot.”
Case irrelevant	Pertaining to noncase-relevant topics (eg, about another procedure or patient, non–work related)		“Is the next patient ready?”
Closed-loop communication steps	Decomposition into types of closed-loop communication		
Call out	Sender transmitting a message		“Can you exchange this instrument?”
Check back	Receiver acknowledging the message		“Yes, I’ll exchange this instrument.”
Closed loop	Sender confirming the correct interpretation or decoding of the message by the receiver		“Thanks.”
Nonverbal communication	Behaviors to complete a task through actions		Handing instrument to surgeon
**Speech metrics (captured with lapel microphones and using Praat speech analysis software)**			
Speech duration, s	Amount of time surgeon spoke	NA	
Speech pitch, Hz	Relative low or high tone perceived by the ear		
Speech intensity, dB	Perceived loudness		
Speech rate, 1/s	Total No. of syllables/s		
**Proximity metrics (captured with lapel microphones)**			
Close	% Of time team member was <1 m away from surgeon	NA	
Near	% Of time team member was 1-3 m away from surgeon		
Far	% Of time team member was >3 m away from surgeon		

Abbreviation: NA, not applicable.

^a^ Five types from content coding, adapted from literature.^5^

**Figure.**
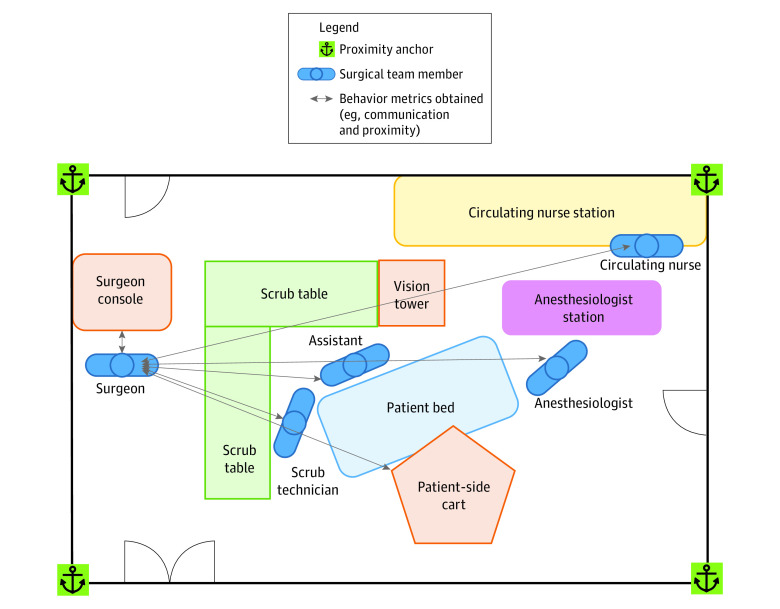
Communication, Speech, and Proximity of Surgical Team Member Location

Surgical teams performing robotic-assisted surgeries (RAS) from general, colorectal, and urology specialties were recruited. After surgeons provided written consent, sensors and video were captured during RAS. Data were segmented into 5 phases: patient-side cart docking, 5 minutes before the critical phase, critical phase, 5 minutes after the critical phase, and 10 minutes before patient-side cart undocking.

After multicollinearity was checked, metrics with significant association (2-sided hypothesis tests, α = .05) with NOTSS scores were identified with Pearson correlations (hypothesis 1) in RStudio version Rv1.3.1093 (RStudio, PBC) (Hmisc package). Two-sided *P* < .05 indicated statistical significance. Predictors of exemplar NTS behavior (highest NOTSS score; hypothesis 2) were modeled with linear and nonlinear machine-learning algorithms (validated with 3-fold cross validation; details in the eAppendix in the Supplement).

## Results

Thirty-four cases were observed, with the participation of 16 nurses (6 men [38%]; 10 women [63%]), 12 residents or fellows (6 men [50%]; 6 women [50%]), 11 anesthesiologists (7 men [64%]; 4 women [36%]), and 4 surgeons (4 men [100%]). Surgeons had a mean (SD) 15 (16) years of experience and performed RAS at least 4 hours per week. Surgeons’ mean (SD) overall NOTSS score was 3.45 (0.43) (1 = poor, 4 = exemplar^4^). The interrater reliability for the NOTSS score was moderate (0.58, based on 25 cases).

Communication by all team roles except for the surgical assistant was associated with NOTSS scores; metrics from communication types (request [*r* = 0.21-0.30; *P* = .004-.04], confirmation [*r* = 0.24-0.27; *P* = .009-.02], and question [*r* = 0.21-0.28; *P* = .006-.049]), closed-loop communication steps (call out, *r* = 0.22-0.25, *P* = .02-.03; closed loop, *r* = 0.36, *P* = .015), and nonverbal communication (*r* = 0.22-0.26; *P* = .012-.03) were significantly associated. Speech metrics (duration [*r* = 0.43; *P* < .001] and pitch [*r* = 0.40; *P* < .001]) were positively associated. Finally, time spent near the surgeon by the surgical assistant (*r* = 0.43; *P* = .04) and circulating nurse (*r* = 0.43; *P* = .009) was positively associated with higher NOTSS scores.

Using the 19 metrics identified from hypothesis 1, random forest nonlinear model (best model) distinguished exemplar from nonexemplar NOTSS scores during RAS with 77% accuracy (hypothesis 2).

## Discussion

Objective communication, speech, and proximity metrics can capture NTS behaviors in the operating room (eg, detection of less frequent communication with team members outside the sterile field negatively influenced NTS). Surgeons measured with increased closed-loop communication and communication frequency with anesthesiologists and nurses had higher NOTSS scores; these metrics potentially captured behaviors surgeons use to overcome the distance separation from the bedside team during RAS.

Nonverbal communication metrics between surgeons with both circulating nurses and technicians were also associated with NOTSS scores, which may indicate anticipation during tasks such as instrument exchange, previously identified to contribute to team effectiveness.^6^ Nonlinear behavior metric models estimated NOTSS scores better than linear models, suggesting that there may be optimal ranges of behaviors, such as maintaining appropriate loudness instead of consistently increasing or decreasing speech intensity.

Study limitations include moderate interrater reliability, missing sensor data, small single-institution sample, and inclusion of male surgeons only. These limitations indicate directions for future work.

In conclusion, this work presents a starting point for research on sensor-based metrics during RAS to objectively assess NTS. With additional supporting evidence, such sensor-based metrics could be used for effective team performance assessment in the operating room for developing personalized training for team members and to improve patient safety.

## Notes

Supplement. **eAppendix.** Expansion of Statistical Modeling Methods
