# Supplementary material for: Measurement of Nontechnical Skills During Robotic-Assisted Surgery Using Sensor-Based Communication and Proximity Metrics
Source: JAMA Netw Open. 2021 Nov 2;4(11):e2132209. doi: 10.1001/jamanetworkopen.2021.32209 (PMC8564574; doi:10.1001/jamanetworkopen.2021.32209)
Supplement: Supplement. — eAppendix. Expansion of Statistical Modeling Methods [file jamanetwopen-e2132209-s001.pdf]

Supplemental Online Content

Cha JS, Athanasiadis D, Anton NE, Stefanidis D, Yu D. Measurement of nontechnical skills during robotic-assisted surgery using sensor-based communication and proximity metrics. *JAMA Netw Open*. 2021;4(11):e2132209. doi:10.1001/jamanetworkopen.2021.32209

eAppendix. Expansion of Statistical Modeling Methods

This supplemental material has been provided by the authors to give readers additional information about their work.

© 2021 Cha JS et al. *JAMA Network Open.*

eAppendix. Expansion of Statistical Modeling Methods

Missing sensor data (up to 62% for some variables) was addressed by imputation (Multivariate Imputation by Chained Equations [mice] package, v3.13.0). Machine learning algorithms used in this study include Linear Discriminate Analysis (LDA), k-Nearest Neighbors (kNN), Support Vector Machine (SVM) with polynomial kernel, and Random Forest (RF) in RStudio (caret package, v6.0.86). Twenty percent of the data was randomly selected and used as the independent testing set. In the 80% training data, three-fold cross-validation was performed with an 80%-20% split in each fold.

This supplemental material has been provided by the authors to give readers additional information about their work.

© 2021 Cha JS et al. *JAMA Network Open.*
